# Work, Stress and Hidden Alcohol Harm: A National Analysis of Stress-Driven Drinking in Full-Time Workers

**DOI:** 10.1192/bjo.2026.11214

**Published:** 2026-06-30

**Authors:** Josiah EA Baiden, David McLaughlan, Anthony Eadon, Aaron Brown, Faith Matcham

**Affiliations:** 1 University of Sussex, Brighton, United Kingdom; 2 Clean Slate Clinic, London, United Kingdom

## Abstract

**Aims:**

Alcohol use in the workplace is typically addressed through impairment-focused policies or reactive disciplinary responses. Yet national data suggest a deeper, systemic relationship between work-related stressors and higher-risk alcohol consumption. The aim of this work is to quantify the prevalence of higher-risk alcohol use among full-time workers and investigate the role of work-related stressors and workplace-specific barriers in shaping drinking behaviour and help-seeking patterns.

**Methods:**

Data from a population survey of 2,037 UK adults (mean age=48.77 years, standard deviation=17.74; 1,066 female [52.33%], 971 male [47.67%]) were analysed to examine the prevalence of higher-risk drinking among full-time workers, demographic distribution, attribution of drinking to life stressors, and work-specific barriers that inhibit help-seeking.

**Results:**

Higher-risk alcohol use was substantially elevated among full-time workers (30.8%), second only to students, and distributed across all income levels, including 40% earning £50,000+. Only 9.6% of this group self-identified as heavy drinkers. Work-related pressures emerged as a core driver: 25.7% of higher-risk workers cited work stress, nearly double the rate among all drinkers (14.3%), with additional attribution to cost-of-living pressures, loneliness, and remote working (2.4× more common than in the general sample).These findings support a model in which alcohol use reflects a stress-response pattern rather than individual pathology. Workplace structures also shaped barriers to accessing support: concerns about career impact (17.8%) and difficulty taking time off work (17.1%) featured prominently among higher-risk adults, indicating that traditional clinic-based services are poorly aligned with the needs of working populations.

**Conclusion:**

Workforce alcohol harm in the UK is widespread, socially invisible, and tightly linked to occupational and economic stressors. Reliance on self-disclosure, performance deterioration, or manager-led referral will systematically miss the majority of affected workers. These findings emphasise the importance of incorporating AUDIT-C screening into occupational health processes and routine workplace wellbeing assessments, ensuring that alcohol-related risk is identified proactively rather than reactively. Alcohol use should be recognised as a meaningful indicator of occupational mental health, considered alongside other markers such as workplace stress, sleep disruption, and burnout. It is also essential to commission flexible, digital, or remote care pathways that minimise career-related stigma and reduce the need for employees to take time away from work in order to access support. Finally, workplace alcohol interventions should be reframed away from an emphasis on individual responsibility and instead approached as part of a broader organisational mental health strategy, acknowledging the role of workplace conditions in shaping alcohol-related risk.

